# Digital Information Approach through Social Media among Gen Z and Millennials: The Global Scenario during the COVID-19 Pandemic

**DOI:** 10.3390/vaccines10111822

**Published:** 2022-10-28

**Authors:** Lorenzo Blandi, Michela Sabbatucci, Giulia Dallagiacoma, Federica Alberti, Paola Bertuccio, Anna Odone

**Affiliations:** 1 School of Public Health, Department of Public Health, Experimental and Forensic Medicine University of Pavia, 27100 Pavia, Italy; 2 Italian National Institute of Health, Department Infectious Diseases, 00161 Rome, Italy

**Keywords:** COVID-19, digital information, social media, survey, Gen Z, Millennials

## Abstract

An infodemic represents a concern for public health, influencing the general population’s perceptions of key health issues. Misinformation is rapidly spread by social media, particularly among young generations. We used data from the WHO “Social Media and COVID-19” study, which was conducted in 24 countries worldwide on over 23,000 subjects aged 18–40 years, to explore Generation Z and Millennials’ models for health-information-seeking behaviors on social media. We summarized data on the most used sources of information, content of interest, and content sharing, as well as the sentiment toward the infodemic, through descriptive statistics and Chi-square test to verify the differences between groups. Among the survey respondents, 9475 (40.3%) were from high-income countries (HIC), 8000 (34.1%) from upper-middle-income countries (UMIC), and 6007 (25.6%) from lower-middle-income countries (LMIC). Social media were the most used sources of information to retrieve news on COVID-19 disease (about 79% in HIC, 87% in UMIC, and 90% in LIC) and the COVID-19 vaccine (about 78% in HIC and about 88% in UMIC and LIC). More than a half of the young respondents declared that they pay attention to scientific contents (about 51% in HIC, 59% in UMIC, and 55% in LMIC). Finally, most young participants reported feeling overwhelmed by the infodemic. However, this sentiment did not stop them from seeking information about COVID-19. Our findings highlight the importance of shaping public health interventions and campaigns on social media platforms and leveraging scientific contents. Public health authorities should work also on strategies to improve the digital literacy of the population as a driving force to empower them and achieve better health outcomes.

## 1. Introduction

The COVID-19 pandemic came together with an infodemic, which was as great a threat to public health as the virus itself. The World Health Organization (WHO) referred to an infodemic as excess information including false or misleading information in digital and physical environments during an acute public health threat [1]. The dynamics of information’s transmission changed in recent decades due to the World Wide Web, which overcame the so-called agenda-setting process theory [2,3]. This theory states that the news media significantly influence our perceptions of what the most salient issues of the day are [4] and, in shaping political reality, act as an intermediary between information and citizens [3]. Social media platforms have been recognized as important tools for health-promoting practices in public health, and their use is widespread among the public. Currently, social media are consistently replacing traditional media and are radically changing the mechanisms by which people access information, exposing them to both true and fake news without any intermediation, so the intersection of the COVID-19 pandemic and the infodemic raised many public health issues to solve [5]. On one hand, medical misinformation causes a significant health toll around the world, contributing to the use of unproven treatments, nonadherence to mitigation measures, and a high level of vaccine hesitancy [6,7]. During the pandemic, people were overwhelmed by different sources of information, especially by social media [8], while different health recommendations and policy responses to SARS-CoV-2 were set across the world [9,10,11]. On the other hand, social media users tend to confine their attention to a limited number of virtual spaces, thus determining a sharp community structure among news outlets and exposing them only to similar opinions [12,13]. Indeed, aggregation in homophilic clusters of users dominates online dynamics, which show clear differences between several social media platforms [14]. This aggregative phenomenon leads to the so-called echo chamber effect, causing poor digital interactions between ever-greater polarized parties [15,16,17]. At the same time, the social-media-based infodemic caused some difficulties for people to self-orient among COVID-19-related news, and it caused mental health concerns [18]. In fact, greater apprehension was expressed about the younger people from 10 to 25 years old [19,20,21]. In this heterogeneous context, young people who belong to Generation Z, also known as Gen Z (born 1997–2012), and Millennials (born 1981–1996) heavily relied on social media as a source of information [22]. Gen Z frequently search for and retrieve informative content through social media [23] but are highly distrustful to this platform [24]. However, possible cross-cultural differences in attitudes to seek health-related information exist between individual countries [25], and results depend on country samples. With the purpose to investigate social media usage and the awareness of false news in regard to COVID-19 information among Gen Z and Millennials, a global study [26] involving 24 countries and over 23,000 respondents was conducted during the first pandemic year. This international study also looked at the size of social media networks and how likely unverified information was to be shared. Here, we analyzed data from the global survey, with the aim to provide a descriptive scenario about the use of digital sources among young generations, their approach and attitude toward health-related information, and the sentiment toward the infodemic, with a focus on comparisons across countries. The results of our study can help policy makers, media, trainers, and schools sharpen effective social media usage on health promotion during pandemic times.

## 2. Materials and Methods

We analyzed individual data from the “Social media and COVID-19” study, a large global cross-sectional study that was conducted in 24 countries from all continents between 24th October 2020 and 7th January 2021, to investigate the digital crisis interaction and the infodemic’s aspects among young people aged 18–40 years. This study was conducted by the University of Melbourne, in partnership with Wunderman Thompson (a global marketing communications agency), Pollfish (an online survey platform provider in New York), and the WHO. An online interactive dashboard was created to give general information on the study and free access to the entire dataset [26,27]. A total of 23,482 young respondents were included in the study. The sampling was designed to balance gender and age groups among Gen Z (born between 1997 and 2012) and Millennials (born between 1981 and 1996). A questionnaire was developed at the University of Melbourne and distributed by Pollfish via their mobile devices over the study period. Data were collected through 26 questions on different aspects, including information on the social media platforms used, the most trusted sources of information for COVID-19 and the related vaccine, the attitude towards finding and sharing scientific content, their awareness and attitude towards fake news, who they share information with, and how they respond to mis- or dis-information (available in the Supplementary Material, Supplementary File S1).

We selected 14 out of the 26 questions of the survey, focusing on those characteristics and information that can be useful for vaccination campaigns targeting the vaccination behavior of young generations and relevant public health interventions (i.e., which social media platforms or messaging apps were usually used, the size of the social network, the source trusted for COVID-19 and vaccine-related news, the content considered attracting and shared, awareness of and reaction to false news, and feelings on and reaction to overwhelming information). Therefore, for a characterization of the young participants, besides the variables of gender, age groups, and education level, we also considered the household income. The questions related to which sources were used first, both for COVID-19 news and updates and the related vaccine, included a range of 17 possible answers that were grouped into three categories: (a) national and international media channels; (b) social media content (shared by experts, family and friends, governments and organizations, religious organizations, and actively searched on websites); and (c) conversation with people (i.e., family and friends, co-workers, religious leaders, and educators). In addition, participants were asked about their feelings and level of agreement with statements regarding the government’s handling of COVID-19 and its vaccine as well as with the information received through social media, on an ordinal scale of five answers from “I strongly agree” to “I strongly disagree”. Finally, for additional analyses focusing on participants’ behavior, we considered the questions related to the size of their social network, their awareness of the correctness of the shared information and their behavior toward fake news, engagement with the World Health Organization (WHO) during the COVID-19 crisis, and related motivation.

For comparisons across countries, we classified the 24 countries into three groups according to the World Bank classification of income level: (1) high-income countries, HIC (i.e., Australia, China, France, Italy, Japan, South Korea, Spain, Sweden, the United Kingdom (UK), and the United States of America (USA)); (2) upper-middle-income countries, UMIC (i.e., Argentina, Brazil, Colombia, Mexico, Peru, Russia, South Africa, and Turkey); and (3) lower-middle-income countries, LMIC (i.e., Egypt, India, Indonesia, Morocco, Nigeria, and the Philippines). Whereas, for the agreement questions, given the high variability we observed in the answers within the income groups (i.e., HIC, UMIC, and LMIC), we preferred to show the distributions in each single country rather than the income groups. For data visualization, we decided to show all five possible answers with gradient color bar charts in order to capture the most detailed information available.

We summarized the data using descriptive statistics (i.e., percentages) and tested the differences between groups (HIC vs. UMIC and HIC vs. LMIC) through the Pearson’s chi-square test, using the Bonferroni’s correction for multiple comparisons (therefore, the *p*-value for a significant difference was 0.02, i.e., 0.05 divided by three comparisons). Since the sampling procedure guaranteed similar distributions across countries by sex, age groups, and education level, we did not further investigate these variables in our analysis.

## 3. Results

Out of the overall sample of 23,482 young respondents (age range: 18–40 years), 9475 (40.3%) were from HIC, 8000 (34.1%) from UMIC, and 6007 (25.6%) from LMIC. The distributions of the general characteristics of the subjects across the three groups are shown in Table 1.

The whole survey was gender-balanced, about 32–33% were young (aged 18–24), and 49.7% in HIC, 49.1% in UMIC, and 46.8% in LMIC were highly educated. In addition, 15.9%, 15%, and 20.5% of the young participants indicated they were students in the HIC, UMIC, and LMIC, respectively, while those who indicated they were not employed were 4.7%, 6.3%, and 10.4%, respectively. The percentages of participants who declared they had a household income under $500 were 4.0% in the HIC, 22.3% in the UMIC, and 34.2% in the LMIC, while those with incomes above $15,000 were 4.2%, 1.8%, and 1.0%, respectively.

Table 2 shows the distributions of the variables of our interest, according to income group, along with pairwise comparisons. The social media platform most frequently used by young generations was YouTube in HIC (59.3%) and UMIC (73%), closely followed by Instagram and Facebook. In LMIC, the most frequently used platform was Facebook (76.1%), followed by YouTube (65.3%) and Instagram (59.6%). Smaller shares of respondents in all the three groups used TikTok and Twitter.

The most frequently used source of information for COVID-19-related content was social media in all groups, although with a lower proportion in HIC (78.8%) than in UMIC (86.9%) and LMIC (89.9%). Similar distributions were reported for information regarding the COVID-19 vaccine, as the content provided by social media was the most used source of information. Similar shares of young respondents in the three groups used national and international media as sources of information for both COVID-19 (54.2% in HIC, 60.3% in UMIC, and 59.5% in LMIC) and vaccine-related contents (46.0%, 53.2%, and 53.7%, respectively). The respondents less frequently declared they retrieved information by person-to-person interactions (for COVID-19 news: 39.6% in HIC, 41.7% in UMIC, and 36.6% in LMIC; for vaccine news: 29.8% in HIC, 28.6 in UMIC, and 27.6 in LMIC).

When asked about the characteristics that they pay attention to when checking COVID-19-related content, more than half of the respondents in all country groups reported “scientific” (51.3% in HIC, 59.3% in UMIC, and 54.7% in LMIC), followed by “content that is relevant to me” (37.4% in HIC, 39.8% in UMIC, and 31.5% in LMIC). In UMIC, a larger share of respondents, compared to HIC and LMIC, also reported paying attention to “content that is concerning” (37.8%, 27.2%, and 32.8%, respectively). The presence of a video was more relevant for respondents from LMIC compared to UMIC or HIC (33.5%, 26.6%, and 20.1%, respectively). In addition, respondents from all income groups reported that they are more likely to share “scientific” content, especially in LMIC (50.7% vs. 47.6% in UMIC and 36.4% in HIC). Young people from LMIC more frequently reported sharing content that included a video compared to HIC or UMIC (33.1% vs. 18.8% and 23.7%, respectively).

Supplementary Table S1 shows the distributions of the number of social media friends or followers as well as a selection of six questions on young participants’ behaviors. Participants from LMIC tended to declare a higher number of friends or followers. Overall, most of the young participants made sure that their shared content was correct (all the time in 33.8% of participants in the HIC, 45.8% in the UMIC, and 45% in the LMIC), although people from HIC tended to share content in a lower proportion. Substantial variability in the answers emerged about the reaction to COVID-19, with the most frequent answers being “I ignore the content” in all the income groups (36.4% in the HIC, 36.7% in the UMIC, and 30.7% in the LMIC) and “I report the content” (22.7% in the HIC and 28.1% in the UMIC) or “I comment on the content” (25.7% in the LMIC). A high level of WHO engagement during the COVID-19 crisis emerged in our sample, though there were some countries differences, where higher proportions of young people who declared to actively search, visit, and follow the WHO website and social media where found in LMIC (52–53%) compared to UMIC (38–43%) and HIC (28–35%).

**Figure 1:**
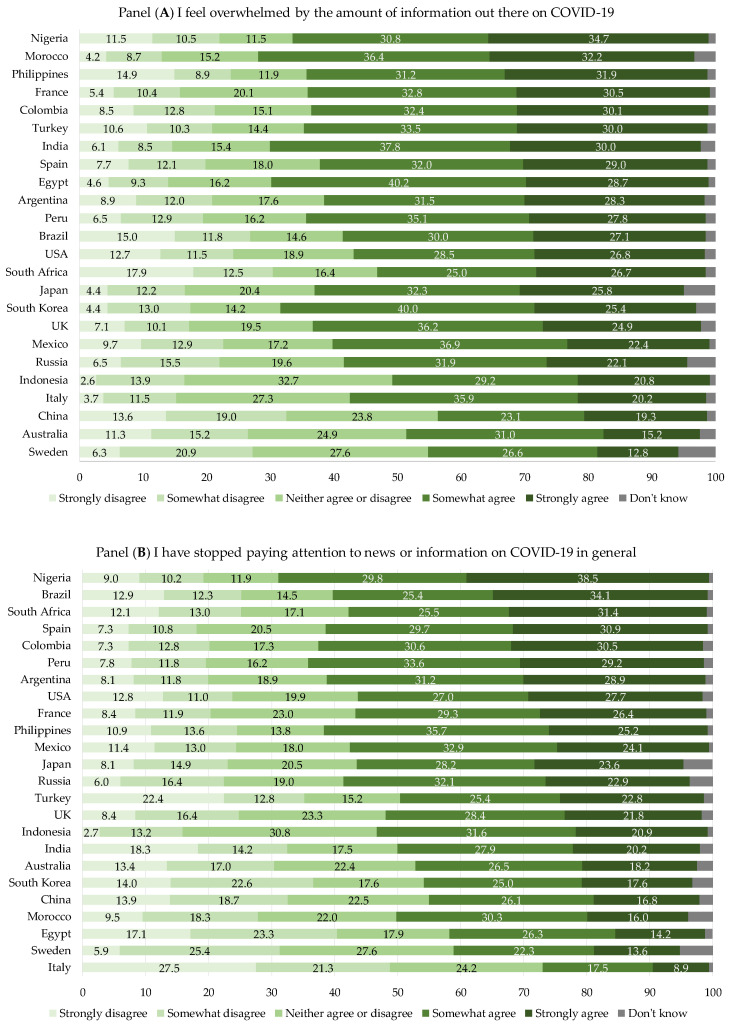

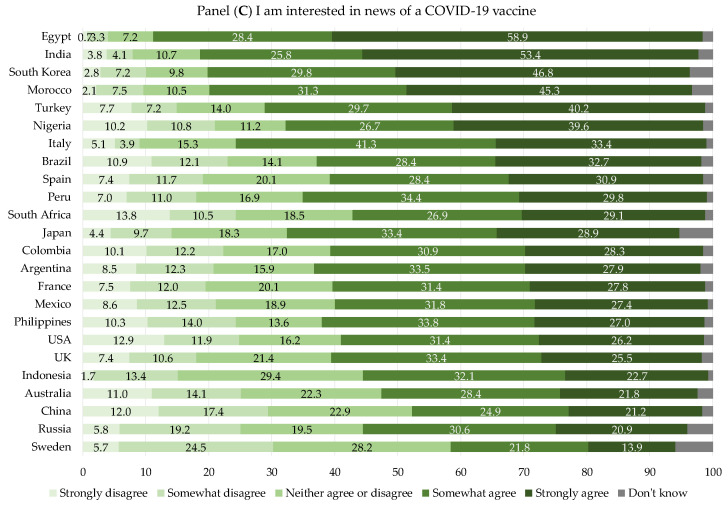
Responses (%) of the 23,482 young respondents to the international survey “Social Media & COVID-19” about the sentiment of agreement with three statements in 24 countries worldwide. Panel (**A**): I feel overwhelmed by the amount of information out there on COVID-19; Panel (**B**): I have stopped paying attention to news and information on COVID-19 in general; Panel (**C**): I am interested in news of a COVID-19 vaccine.

Figure 1 shows the distribution of the sentiment of agreement (from “strongly disagree” to “strongly agree”) with three selected statements by country, including “I feel overwhelmed by the amount of information out there on COVID-19” (panel A), “I have stopped paying attention to news/information on COVID-19 in general” (panel B), and “I am interested in news of a COVID-19 vaccine” (panel C). Countries are ordered by the “strongly agree” response. As for the statement in panel A, the proportion of participants who declared “strongly agree” ranged from 12.8% in Sweden and 15.2% in Australia to 34.7% in Nigeria, with similar results in Morocco (32.2%) and the Philippines (31.9%). On the other hand, the proportion of participants who declared “strongly disagree” was lower in all countries, ranging from 2.6% in Indonesia to 17.9% in South Africa, followed by the Philippines (14.9%) and China (13.6%). For the statement in panel B, the highest percentage of respondents who declared “strongly agree” was reported in Nigeria (38.5%) and Brazil (34.1%), while the lowest percentage was reported in Italy (8.9%). The proportions of young people who declared “strongly disagree” ranged from 2.7% in Indonesia to 27.5% in Italy, with all other countries reporting 5.9–18.3%, except for Turkey (22.4%). For the statement in panel C, wide differences across countries were observed for the “strongly agree” answer, ranging from 13.9% in Sweden to 58.9% in Egypt, followed by 53.4% in India and 46.8% in South Korea. On the other hand, the proportion of participants who reported they “strongly disagree” with this statement was low across all countries, ranging between 0.7% in Egypt and 13.8% in South Africa.

## 4. Discussion

This study provides a global descriptive picture about the health-related information approach through digital sources among young generations, in particular about the most used sources for information about COVID-19 and its vaccine, content of interest, and sharing as well as the sentiment toward the infodemic, in pandemic times. We found that the Gen Z and Millennials preferred to access information through social media platforms (including YouTube, Instagram, Facebook, TikTok, and Twitter as the most used platforms overall), they searched for and shared news that included scientific content as a characteristic of interest. In addition, during the infodemic they felt overwhelmed due to the large amount of information they were exposed to, though this did not influence their attention to COVID-19 news. This global survey provided several insights on the digital information approach through social media among Gen Z and Millennials during the COVID-19 pandemic. Here, we aimed to focus on the most important aspects that might be relevant for policy makers and vaccination information campaigns targeting young generations. Considering that Gen Z represent around 27% and Millennials represent around the 24% of the world population [28], targeting these tech savvy users with specialized campaigns aimed at spreading science-based information could provide a multiplier effect on other age groups and ultimately decrease the impact of the infodemic in pandemic times.

To our knowledge, the Eurobarometer survey, requested by the EU Parliament, also provided a recent picture of European citizens on media habits and trust in different media sources as well as attitudes towards disinformation [29]. As in our study, platforms such as Facebook, Instagram, YouTube, TikTok, and Twitter were the most used social media among the European young generations, although with some differences between Gen Z and Millennials. Specifically, Millennials used Facebook more frequently, while Gen Z used Instagram, YouTube, TikTok, and Twitter. Moreover, Gen Z more frequently preferred reading articles or posts on their social networks than Millennials. On the contrary, Millennials used news websites more frequently as sources of information than Gen Z.

As emerged from previous studies, all the mentioned social media platforms contain a significant proportion of health-related misinformation, disseminating it rapidly and far [30] and causing a high-impact issue for public health [6]. The recent European study [29] reported that over a third of both Gen Z and Millennials declared they were exposed to misinformation and fake news sometime over the week before the survey. The infodemic and its related misinformation lead to different consequences. At the individual level, many people could try unproven treatments against COVID-19, not following official guidelines for therapies recommended by international and national organizations as well as their general practitioner or other health professionals. At a community level, this phenomenon may increase the number of people who do not trust in mitigation measures, questioning the importance of social distancing [26] and the use of safety devices, such as face masks. From a public health perspective, many people faced difficulties in finding evidence-based information in this chaotic digital environment, with the consequence of a high level of vaccine hesitancy and therefore negative impacts for the health of the population. With reference to this, the European survey [29] highlighted that Gen Z and Millennials felt equally confident that they could recognize misinformation when they encountered it. In particular, the European respondents thought that, in that past week, they were exposed to misinformation and fake news with similar proportions between Gen Z and Millennials.

The young generations from our study reported looking for scientific content as well as a requirement to fact-check before sharing it with their own network. Specifically, respondents from UMIC and LIC, more than those from UIC, paid specific attention to scientific COVID-19 content when checking it, while HIC and UMIC participants, more than LIC participants, checked content relevant for their own situation. These results reflect the fact that they are aware of the key characteristic of information to pay attention to in order to undertake the choice recommended by evidence. Conversely, another previous study [22] reported that young people were unlikely to fact-check the content they view on the World Wide Web with a health professional. This situation pointed out fundamental implications to consider. The young generations recognized the importance of science in their health-related decision-making process, but it should be not taken for granted that they recognize whether content is scientific or not. Young generations need to develop adequate digital literacy [31] to be able to understand the key aspects of scientific thinking, and they also need clear and unambiguous scientific information from their social media platforms. Therefore, policymakers and public health professionals should work to meet these population needs, starting from school education [31]. At the same time, they should not follow the sensationalistic trends of the news market. Indeed, concerning content received around half of the attention of scientific content, as the results of our study highlighted.

In addition, COVID-19 was accepted as a new topic in the online debate, as it was considered a relevant content to the health status of social media users. The scientific characteristic and the personal relevance of information are the aspects most preferred by young generations, and these are therefore fundamental for the implementation of communication plans by public health professionals and policymakers. At the same time, the article title plays a crucial role. The European study [29] highlighted that over half of the respondents pointed to this as main attracting factor to click on a news article.

Data on the sentiment toward the infodemic among young generations suggested that they felt overwhelmed by the amount of information on COVID-19 in most countries. As shown in the literature, many young people suffered psychological pressure, depression, and family and relationship anxieties, along with serious economic worries due to the pandemic [32,33]. Focusing on specific countries in this concerning picture, young generations from both UMIC and HIC (Sweden, China, and Australia) were felt to be less overwhelmed by the COVID-19 infodemic (around 40%) than their peers in other countries. Indeed, they did not stop to pay attention to the news about the pandemic. On the other hand, young generations from LMIC (Morocco, Egypt, and India) were the most hit by this infodemic, although they behaved similarly to the UMIC peers, as they did not stop looking for news about the pandemic. Focusing on between-country comparisons, the attitudes of some countries to stop looking for news were not likely proportionally related to the feeling of being overwhelmed by the infodemic.

This study suggests that most of the young generations, who preferred easy-to-access media, felt overwhelmed by the amount of COVID-19 information; in many countries (e.g., Nigeria, Brazil, Spain, and Colombia), they stopped looking for it, while in some others (e.g., Morocco and Egypt) few of them declared that they stopped paying attention to this news, without any evidence of the countries’ income as a driver of the highlighted differences. In addition, young generations from some UMIC (Egypt, India, and Morocco) and HIC (South Korea) were the most interested in news about the COVID-19 vaccine among their global peers, in contrast with other UMIC (Russia and China) and HIC (Sweden and Australia) peers that were the less interested on this topic. These findings suggest that the infodemic did not lead to an addiction or, instead, an indifference of the global young generations to COVID-19 news.

From the global report [26], it also emerged that the WHO and national newspapers, TV, and radio were the first authors of COVID-19 vaccine information, in opposition to the social media communities of friends that were at the bottom of this ranking. However, it is important to underline that this content about the COVID-19 vaccine is provided by social media platforms. Consequently, public health professionals and policymakers should consider this latter virtual environment to reach the young generations.

As strengths, this study highlights the importance of investigating health-related social behaviors before and after public health interventions. The policy makers and the public health professionals must shape their health plans and interventions through the information platforms most used by the target population. Knowledge and updates about the main sources of information are crucial for the commitment of a target population to preventive health measures. Additionally, this study adds a descriptive landscape of the digital information environment among young generations, advancing the knowledge within the field, especially for future vaccination and other public health campaigns.

On the other hand, this study has some limitations. The descriptive nature of this analysis does not allow us to find correlations between variables of interest, and it will be necessary to find other drivers to carry out further and deep analyses. It will also be interesting to focus on the use of social media by the elderly population, who does not have the same level of digital literacy [34] and could trust the news differently and develop anxieties [35], leading to social disparities related to information and service access.

For the public health purpose, it emerged that all national and local authorities and governments must be present on social media platforms, and public health professionals should be trained in their correct use. Indeed, all public health campaigns intended for young generations must consider social media platforms as the main channels to convey prevention and health promotion information to the general audience [36,37].

## 5. Conclusions

Young generations from all over the world, and mostly from UMIC and LIC, preferred social media platforms as providers of news and information, especially YouTube, Instagram, Facebook, TikTok, and Twitter. During the COVID-19 pandemic, they felt overwhelmed by the COVID-19 infodemic, with heterogeneous sentiments across countries, and many of them paid less attention to COVID-19 news. A complex situation emerged, where young generations could not easily orient themselves among the digital communication environment, leading to high-impact issues for public health. However, the young generations recognized the importance of science in their health-related decision-making process, so policymakers and public health professionals should work on strategies that enable young generations to have digital literacy for discerning scientific content, especially during the infodemic. On a cross-national comparison perspective, the absence of evident generalizable information dynamics in this heterogenous and globalized scenario suggests that the national communication environment influenced these dynamics the most, maybe due to political, economic, and social factors. However, interest in the SARS-CoV-2 vaccine remained high and stable, and social media platforms were the main sources to provide information to young generations about these vaccines. It became evident that the national and local authorities and governments must use social media platforms for their public health campaigns and to spread evidence-based scientific content. In addition, public health professionals should be trained in their correct use in order to reach the target population effectively and maximize their efforts.

## Figures and Tables

**Table 1:** General characteristics of the 23,482 young respondents to the international survey “Social Media & COVID-19” by sex, age group, education level, and household income in the three income groups (according to the World Bank classification of income level).

	HIC N = 9475		UMIC N = 8000		LMIC N = 6007	
	*n*	%	*n*	%	*n*	%
Sex						
Females	4782	50.5	3994	49.9	2929	48.8
Males	4693	49.5	4006	50.1	3078	51.2
Age group						
18–24 (Gen Z)	3066	32.4	2635	32.9	2018	33.6
25–40 (Millennials)	6409	67.6	5365	67.1	3989	66.4
Education level						
Low	573	6.0	419	5.2	219	3.6
Intermediate	4193	44.3	2650	33.1	1974	32.9
High	4709	49.7	3931	49.1	2813	46.8
Missing *	-	-	1000	12.5	1001	16.7
Household income ($)						
I am a student	1509	15.9	1200	15.0	1230	20.5
Under 500	379	4.0	1783	22.3	2057	34.2
501 to 1000	610	6.4	1949	24.4	1100	18.3
1001 to 1500	1012	10.7	1078	13.5	355	5.9
1501 to 2500	1683	17.8	630	7.9	204	3.4
2501 to 5000	2001	21.1	311	3.9	108	1.8
5001 to 7500	710	7.5	115	1.4	74	1.2
7501 to 10,000	369	3.9	87	1.1	55	0.9
10,001 to 12,500	174	1.8	85	1.1	53	0.9
12,501 to 15,000	105	1.1	80	1.0	37	0.6
>15,000	396	4.2	145	1.8	63	1.0
I am not employed	450	4.7	506	6.3	624	10.4
Other	77	0.8	31	0.4	47	0.8

HIC: High-income countries; LMIC: Low-middle-income countries; UMIC: Upper-middle-income countries. * No information on education level for Nigeria and South Africa.

**Table 2:** Responses (%) of the 23,482 young respondents to the international survey “Social Media & COVID-19” about the social media platforms usually used, the sources of information regarding COVID-19 and its vaccine, and the characteristics of COVID-19 content and shared content in the three income groups (according to the World Bank classification of income level).

	HIC N = 9475	UMIC N = 8000	LMIC N = 6007	HIC vs. UMIC	HIC vs. LMIC	UMIC vs. LMIC
	%	%	%	*p*-Value *	*p*-Value *	*p*-Value *
Which social media platforms do you usually use? (the top five most frequent)						
YouTube	59.3	73.0	65.3	<0.01	0.32	<0.01
Instagram	59.2	72.3	59.6	<0.01	<0.01	<0.01
Facebook	55.7	71.8	76.1	<0.01	<0.01	<0.01
TikTok	33.9	35.0	20.9	0.13	<0.01	<0.01
Twitter	31.4	39.4	37.3	<0.01	0.01	0.01
Average number of social media platforms (SD)	2.9 (2.0)	3.4 (1.9)	3.0 (2.0)	0.02	0.17	<0.01
For COVID-19 news, information, and updates, to which of the following sources do you go to first?						
National or international traditional media (newspapers, television, or radio, including websites)	54.2	60.3	59.5	<0.01	<0.01	0.34
Content provided by social media	78.8	86.9	89.9	<0.01	<0.01	<0.01
Content shared person to person	39.6	41.7	36.6	0.01	<0.01	<0.01
When a vaccine becomes available, which of the following sources would you look to first for information?						
National or international traditional media (newspapers, television, or radio, including websites)	46.0	53.2	53.7	<0.01	<0.01	0.56
Content provided by social media	78.0	88.3	88.7	<0.01	<0.01	0.49
Content shared person to person	29.8	28.6	27.6	0.08	<0.01	0.22
When checking COVID 19 content, I pay specific attention to COVID-19 content that: (the top five most frequent answers)						
is scientific	51.3	59.3	54.7	<0.01	<0.01	<0.01
is relevant to me	37.4	39.8	31.5	<0.01	<0.01	<0.01
includes/is an article	36.2	37.4	31.2	0.10	<0.01	<0.01
is concerning	27.2	37.8	32.8	<0.01	<0.01	<0.01
includes/is a video	20.1	26.6	33.5	<0.01	<0.01	<0.01
I am most likely to share content with my networks that: (the top five most frequent answers)						
is scientific	36.4	47.6	50.7	<0.01	<0.01	<0.01
is relevant to me	32.1	43.6	34.7	<0.01	<0.01	<0.01
includes/is an article	25.8	29.1	31.3	<0.01	<0.01	0.01
is concerning	23.3	34.0	29.4	<0.01	<0.01	<0.01
includes/is a video	18.8	23.7	33.1	<0.01	<0.01	<0.01

HIC: High-income countries; LMIC: Low-middle-income countries; UMIC: Upper-middle-income countries; SD: standard deviation. * *p*-value for the comparison test (i.e., chi-square test or *t*-test for the number of platforms usually used). Since the Bonferroni’s correction for multiple comparisons was adopted, a *p*-value < 0.02 (0.05/3) indicates significant differences among pairs.

## Data Availability

All data can be downloaded at: https://covid19-infodemic.com/#resources (accessed on 26 September 2022).

## Supplementary Materials

The following supporting information can be downloaded at: https://www.mdpi.com/article/10.3390/vaccines10111822/s1, Table S1: Percentages of young respondents about selected behaviors, by geographical area (according to the World Bank classification by income level); Supplementary File S1: Questionnaire.
