# Supplementary material for: Digital Information Approach through Social Media among Gen Z and Millennials: The Global Scenario during the COVID-19 Pandemic
Source: Vaccines (Basel). 2022 Oct 28;10(11):1822. doi: 10.3390/vaccines10111822 (PMC9696549; doi:10.3390/vaccines10111822)
Supplement: Supplementary file 1 [file vaccines-10-01822-s001.zip › Supplementary File S1 - Questionnaire.pdf]

Supplemental material – Questionnaire

Q1 In today's global COVID19 crisis, some people are concerned that they are at risk of infection while others are not -- how concerned are you? (SingleSelection) (Screening question)

- Very concerned
- Somewhat concerned
- Not concerned at all
- I don't care about COVID-19
- I don't know what is COVID-19

Q2 When considering the broader impacts of the COVID-19 crisis - what are you specifically worried about? Please select all that apply. (MultipleSelection)

- Friends and family members getting infected
- Access to education
- Employment uncertainty
- Facing financial constraints
- Economy crashing
- Increasing political tensions
- Significant change of my lifestyle
- Losing touch with my social community
- Not being able to access healthcare
- Uncertainties regarding residency status
- Not being able to travel/ visit friends and family
- Discrimination
- Running out of food
- My mental health
- I am not worried about the broader impacts of the COVID-19 crisis
- Other

Q3 Which social media platforms or messaging apps do you usually use? Please select all that apply. (MultipleSelection)

- Facebook
- Twitter
- Instagram
- TikTok
- WeChat
- LinkedIn
- Tumblr
- WhatsApp
- Snapchat
- Pinterest
- Reddit
- YouTube
- Vimeo
- Discord
- Telegram

- Facebook Messenger
- Skype
- Zoom
- House Party
- Viber
- QZone
- Tencent QQ
- iMessage
- YY
- VK
- Skyrock
- Quora
- Weibo
- Other

Q4 How many friends or followers do you have across your social media? (SingleSelection)

- Under 50
- 50 to 99
- 100 to 249
- 250 to 499
- 500 to 999
- 1,000 to 5,000
- Over 5,000

Q5 For COVID-19 news, information and updates, to which of the following sources do you go to first? (MultipleSelection)

- National newspapers, television and radio (including websites and streaming)
- International newspapers, television and radio (including websites and streaming)
- Social media content by traditional media (e.g. newspapers, television, radio)
- Social media content by my government
- Social media content by the World Health Organization (WHO)
- Social media content by science and health experts
- Social media content by celebrities and influencers
- Social media content by alternative movement leaders
- Social media content by sources I cannot identify
- Social media content by family
- Social media content by friends
- Actively searching using search sites (such as Google, Baidu)
- Friends (as part of a personal conversation)
- Family (as part of a personal conversation)
- Educators (as part of a personal or academic conversation)
- Religious leaders (as part of a personal or religious conversation)
- Co-workers (as part of a personal or workplace conversation)
- Other

Q6 And which of the following sources do you then use to learn more about this information? Please select all that apply. (MultipleSelection)

- National newspapers, television and radio (including websites and streaming)
- International newspapers, television and radio (including websites and streaming)
- Social media content by traditional media (e.g. newspapers, television, radio)
- Social media content by my government
- Social media content by the World Health Organization (WHO)
- Social media content by science and health experts
- Social media content by celebrities and influencers
- Social media content by alternative movement leaders
- Social media content by sources I cannot identify
- Social media content by family
- Social media content by friends
- Actively searching using search sites (such as Google, Baidu)
- Friends (as part of a personal conversation)
- Family (as part of a personal conversation)
- Educators (as part of a personal or academic conversation)
- Religious leaders (as part of a personal or religious conversation)
- Co-workers (as part of a personal or workplace conversation)
- Other

Q7 When a vaccine becomes available, which of the following sources would you look to first for information? (MultipleSelection)

- National newspapers, television and radio (including websites and streaming)
- International newspapers, television and radio (including websites and streaming)
- Social media content by traditional media (e.g. newspapers, television, radio)
- Social media content by my government
- Social media content by the World Health Organization (WHO)
- Social media content by science and health experts
- Social media content by celebrities and influencers
- Social media content by alternative movement leaders
- Social media content by sources I cannot identify
- Social media content by family
- Social media content by friends
- Actively searching using search sites (such as Google, Baidu)
- Friends (as part of a personal conversation)
- Family (as part of a personal conversation)
- Educators (as part of a personal or academic conversation)
- Religious leaders (as part of a personal or religious conversation)
- Co-workers (as part of a personal or workplace conversation)
- Other

Q8 When it comes to trust in sources, people have different opinions. Please rank the sources based on your level of trust from the list below: (MatrixSingleSelection)

	Highly trust	Trust	Somewhat trust	Don't trust	Don't know
Newspapers (including websites, streaming and information these organizations have shared on social media)					
Television (including websites, streaming and information these organizations have shared on social media)					
World Health Organization (WHO)					
Science and health experts					
Family					
Friends					
Educators					
Radio (including websites, streaming and information these organizations have shared on social media)					

Q9 When it comes to trust in sources, people have different opinions. Please rank the sources based on your level of trust from the list below: (MatrixSingleSelection)

	Highly trust	Trust	Somewhat trust	Don't trust	Don't know
Alternative movement leaders					
My government					
Religious leaders					
Co-workers					
Top results on search sites (e.g. Google, Baidu)					
My social media community					
Information from messaging Apps					

Q10 When checking COVID 19 content - I pay specific attention to COVID-19 content which ... Please select all that apply. (MultipleSelection)

- includes/is an image
- includes/is an article
- includes/is a video
- includes/is a meme
- includes/is an audio clip
- includes/is an infographic
- is humorous
- is concerning
- is scientific
- has a lot of shares/likes/retweets
- tells a story
- creates emotional reaction
- is related to an influencer or celebrity
- is relevant to me
- Other
- I don't pay attention to COVID19 content

Q11 I most likely share content with my networks that ... Please select all that apply. (MultipleSelection)

- includes/is an image
- includes/is an article
- includes/is a video
- includes/is a meme
- includes/is an audio clip
- includes/is an infographic
- is humorous
- is concerning
- is scientific
- has a lot of shares/likes/retweets
- tells a story
- creates emotional reaction
- is related to an influencer or celebrity
- is relevant to me
- I don't pay attention to COVID19 content
- Other
- I don't pay attention to COVID19 content

Q12 The term 'fake news' has been used a lot recently. How aware are you of the fact that COVID-19 information on social media/messaging apps could be false? I am ... (SingleSelection)

- very aware
- somewhat aware
- unaware
- I don't care whether content is real or fake

Q13 You said that you don’t care whether content is real or fake, please tell us why (OpenEnded)

-

Q14 We all share a lot of information on social media/messaging apps these days - do you make sure that information is correct before sharing? (SingleSelection)

- All of the time
- Most of the time
- Sometimes
- Never
- I don't share content

Q15 Have you ever shared something on social media/messaging apps that you later found out was incorrect? (SingleSelection)

- Yes
- No
- I don’t know
- I don't share content on social media/messaging apps

Q16 Did you later share a correction or delete? (SingleSelection)

- Yes
- No

Q17 How do you react to COVID-19 information - shared by others on social media/messaging apps - that you know is false ? (SingleSelection)

- I comment on the content
- I report the content
- I unfollow the person who posted it
- I ignore the content
- I share the content
- I don't know

Q18 Overall, how do you feel about the existence of 'fake' information regarding COVID-19 on social media/messaging apps? (SingleSelection)

- I am very concerned
- I am somewhat concerned
- I am not concerned
- I find it interesting
- I don't know

Q19 How strongly do you agree or disagree with the following statements, when it comes to COVID-19? (MatrixSingleSelection)

	Strongly agree	Somewh at agree	Neither agree or disagree	Somewhat disagree	Strongly disagree	I don't know
I feel overwhelmed by the amount of information out there on COVID-19						
All information on social media is the same to me						
I feel the media is not telling me everything						
I have stopped paying attention to news and information on COVID-19 in general						
I feel like my government is not giving me the full picture when it comes to COVID-19						
I feel my government is not doing enough to control the pandemic						
I feel like my government is overreacting when it comes to COVID-19						
I am interested in news of a COVID-19 vaccine						

Q20 How strongly do you agree or disagree with the following statements, when it comes to COVID-19? (MatrixSingleSelection)

	Strongly agree	Somewh at agree	Neither agree or disagree	Somewhat disagree	Strongly disagree	I don't know
I believe that when we have the vaccine things will go back to normal						
I am optimistic about the future						
I believe that COVID-19 content is biased						
Social distancing and hygiene directives are not really necessary						

Q21 Considering your engagement with COVID-19 from months ago to now: What COVID-19 content were you interested in at the start of the pandemic? (MultipleSelection)

- International updates
- National/local updates
- WHO health directives
- Health directives of my government
- International travel restrictions
- National/local travel restrictions
- Information about a vaccine
- Stories about personal experiences
- The opinions of celebrities/influencers
- I was not interested in COVID-19 content at the start of the pandemic

Q22 What COVID-19 content are you interested in now? (MultipleSelection)

- International updates
- National/local updates
- WHO health directives
- Health directives from my government
- International travel restrictions
- National/local travel restrictions
- Information about a vaccine
- Stories about personal experiences
- The opinions of celebrities/influencers
- I am not interested in COVID-19 content now

Q23 How have you engaged with the World Health Organization (WHO) so far during the COVID-19 crisis? (MultipleSelection)

- I actively search for WHO health directives
- I visit the WHO website
- I follow the WHO on social media
- I do not engage with WHO
- Other

Q24 Why have you not engaged with the World Health Organization (WHO) during the COVID-19 crisis? Please select all that apply. (MultipleSelection)

- I don't know what WHO is
- I do not engage with WHO content because I do not trust it
- I do not engage with WHO content because I have been using other sources
- I don’t know how to find WHO information
- Other

Q25 How do you think the World Health Organization (WHO) could improve communication about the COVID 19 crisis ? (OpenEnded)

-

Q26 One last question, what is monthly Household income? (SingleSelection)

- I am a student
- Under $500 USD
- $501 to $1,000 USD
- $1,001 to $1,500 USD
- $1,501 to $2,500 USD
- $2,501 to $5,000 USD
- $5,001 to $7,500 USD
- $7,501 to $10,000 USD
- $10,001 to $12,500 USD
- $12,501 to $15,000 USD
- $15,000 USD+
- I am not currently employed
