# Supplementary material for: Digital Information Approach through Social Media among Gen Z and Millennials: The Global Scenario during the COVID-19 Pandemic
Source: Vaccines (Basel). 2022 Oct 28;10(11):1822. doi: 10.3390/vaccines10111822 (PMC9696549; doi:10.3390/vaccines10111822)
Supplement: Supplementary file 1 [file vaccines-10-01822-s001.zip › Supplementary Table S1.pdf]

Table S1. Percentages of young respondents about selected behaviors, by geographical area (according to the World Bank classification by income level).

	HIC N = 9475	UMIC N = 8000	LMIC N = 6007	HIC *vs* UMIC	HIC *vs* LMIC	UMIC *vs* LMIC
	%	%	%	*p*-value*	*p*-value*	*p*-value*
**How many social media friends or followers**				<0.01	<0.01	<0.01
Under 50	14.9	11.4	11.4			
50 to 99	12.0	10.1	9.5			
100 to 249	21.1	17.1	16.4			
250 to 499	19.5	19.1	15.8			
500 to 999	16.1	18.9	19.0			
1000 to 5000	12.9	19.0	22.3			
Over 5000	3.5	4.4	5.6			
**We all share a lot of information on social media/messaging apps these days - do you make sure that information is correct before sharing?**				<0.01	<0.01	<0.01
I don't share content	8.4	4.5	4.7			
Sometimes	18.1	14.4	16.7			
Most of the time	39.7	35.3	33.6			
All of the time	33.8	45.8	45.0			
**Have you ever shared something on social media/messaging apps that you later found out was incorrect?**				<0.01	<0.01	<0.01
I don't share content on social media/messaging apps	8.4	5.2	5.8			
I don't know	17.9	11.1	9.5			
No	38.1	46.1	44.3			
Yes	35.5	37.6	40.4			
**Did you later share a correction or delete?**				0.16	0.01	0.21
No	4.5	4.9	4.8			
Yes	27.4	32.8	35.7			
Missing	*68.1*	*62.3*	*59.5*			
**How do you react to COVID?**				<0.01	<0.01	<0.01
I ignore the content	36.4	36.7	30.7			
I report the content	22.7	28.1	22.0			
I comment on the content	16.8	17.5	25.7			
I unfollow the person who posted it	8.8	8.2	9.1			
I share the content	6.9	5.6	9.2			
I don't know	8.3	3.8	3.2			
**How have you engaged with the World Health Organization (WHO) so far during the COVID-19 crisis?**						
I actively search for WHO health directives	26.7	35.8	42.7	<0.01	<0.01	<0.01
I visit the WHO website	34.5	42.8	51.6	<0.01	<0.01	<0.01

I follow the WHO on social media	27.6	38.3	53.1	<0.01	<0.01	<0.01
I do not engage with WHO	35.8	22.7	14.0	<0.01	<0.01	<0.01
**Why have you not engaged with the World Health Organization (WHO) during the COVID-19 crisis?**						
I don't know what WHO is	3.4	1.6	0.8	<0.01	<0.01	<0.01
I do not engage with WHO content because I do not trust it	6.7	6.9	2.8	0.57	<0.01	<0.01
I do not engage with WHO content because I have been using other sources	13.8	8.5	5.0	<0.01	<0.01	<0.01
I don't know how to find WHO information	7.8	3.9	3.2	<0.01	<0.01	0.02

HIC: High income countries; LMIC: Low-middle income countries; UMIC: Upper-middle income countries. *p-value for the comparison test (i.e., Chi-square test or t-test for the number of platforms usually used). Since Bonferroni's correction for multiple comparisons is adopted, a p-value < 0.02 (0.05/3) indicates significant differences among pairs.
